# Iatrogenic Superficial External Pudendal Artery Pseudoaneurysm: Treatment with Doppler US-Guided Compression

**DOI:** 10.5812/iranjradiol.7228

**Published:** 2014-05-15

**Authors:** Oktay Algin, Assanaly Mustafayev, Evrim Ozmen

**Affiliations:** 1 Department of Radiology, Ataturk Hospital, Bilkent, Ankara, Turkey; 2 National MR Research Center (UMRAM), Bilkent University, Ankara, Turkey; 3 Department of Radiology, Ahmet Yesevi University, Turkestan, Kazakhstan; 4 Department of Radiology, Cerrahpasa Medical School Hospital, Istanbul University, Istanbul, Turkey

**Keywords:** Angiography, Catheterization, Aneurysm, False, Femoral Artery

## Abstract

Pseudoaneurysms rarely occur as a serious complication following incomplete hemostasis of an arterial puncture site. As a result of the increase in diagnostic and therapeutic angiography, the frequency of iatrogenic pseudoaneurysm has increased as well. Iatrogenic pseudoaneurysms associated with angiographic catheterization occur most commonly in the common femoral artery. Here we report a case of iatrogenic superficial external pudendal artery (SEPA) pseudoaneurysm following cardiac catheterization, which was diagnosed with Doppler ultrasound (US) and multidetector computed tomographic angiography (MDCTA) before Doppler US-guided compression therapy. To the best of our knowledge, iatrogenic SEPA pseudoaneurysm, which is an unusual vessel location for pseudoaneurysm occurrence, has not been reported in the literature. In patients in whom anticoagulant-thrombolytic therapy or therapeutic catheterization with larger sized sheath is planned, determination of the precise localization of arterial puncture site is important for the prevention of iatrogenic pseudoaneurysm development. Arterial puncture guided with Doppler US might reduce complications. When suspected, MDCTA is useful in the diagnosis and demonstration of iatrogenic pseudoaneurysms. Treatment of US-guided compression should be the first choice for iatrogenic pseudoaneurysms. Interventional radiologists and cardiologists should have enough experience about the catheterization complications and their treatment in order to decrease the morbidity and mortality related to the intervention.

## 1. Introduction

Iatrogenic pseudoaneurysm (IPA) is an uncommon, but well-defined, and important complication following angiographic studies (1). Pseudoaneurysm occurs from a post-puncture defect in the arterial wall with an extravasation of blood into the surrounding inguinal tissue forming a pulsatile hematoma (2). There are some factors that could increase the risk for IPA such as improper access site, multiple punctures, and insufficient post-procedural compression (3). Moreover, with the increasing number of larger sized angiographic sheath use, prolonged procedure duration, and thrombolytic-anticoagulant therapy, the incidence of IPA has increased recently (4). Therefore, the incidence of IPA development is higher in patients in whom catheterization for therapeutic angiography is performed (3).

In this case report, we present the development of IPA in the superficial external pudendal artery (SEPA) of a patient in whom therapeutic angiography in the left groin was performed. To the best of our knowledge, this is the first report in the literature concerning iatrogenic SEPA pseudoaneurysm due to angiographic catheterization.

## 2. Case Presentation

A 52-year-old male patient was admitted to our interventional radiology unit with pain, swelling and color change in the left inguinal region following therapeutic angiography. Laboratory tests showed progressive reduction in hemoglobin levels. In the diagnostic angiography that was performed two days earlier, partial occlusion in the left anterior descending artery was observed and 1 day before the patient was referred to us, he had undergone percutaneous transluminal coronary angioplasty with stent placement. The patient was under thrombolytic-anticoagulant therapy at the time of angioplasty. He had been smoking one-pack-cigarette/day for the last 40 years and he had high blood pressure. No additional important information was detected in his medical history.

In the gray scale ultrasonographic examination, a 43×26 mm pulsatile cystic lesion was observed in the left groin. Doppler ultrasound (US) examination showed “to and fro blood flow” inside the lesion. Based on these findings, the patient was diagnosed as IPA, but IPA and the morphology of its neck could not be demonstrated clearly with US. To detect the morphology, the origin, and the neck of IPA, multidetector computed tomographic angiography (MDCTA) was recommended. US-guided compression was done for 20 minutes, as well. Following compression treatment, a thrombus that formed one third of the size of the IPA was detected. The patient was recommended to sleep and bed rest in the supine position, and pressure was applied on his left groin and he was observed for one day.

**Figure 1.**
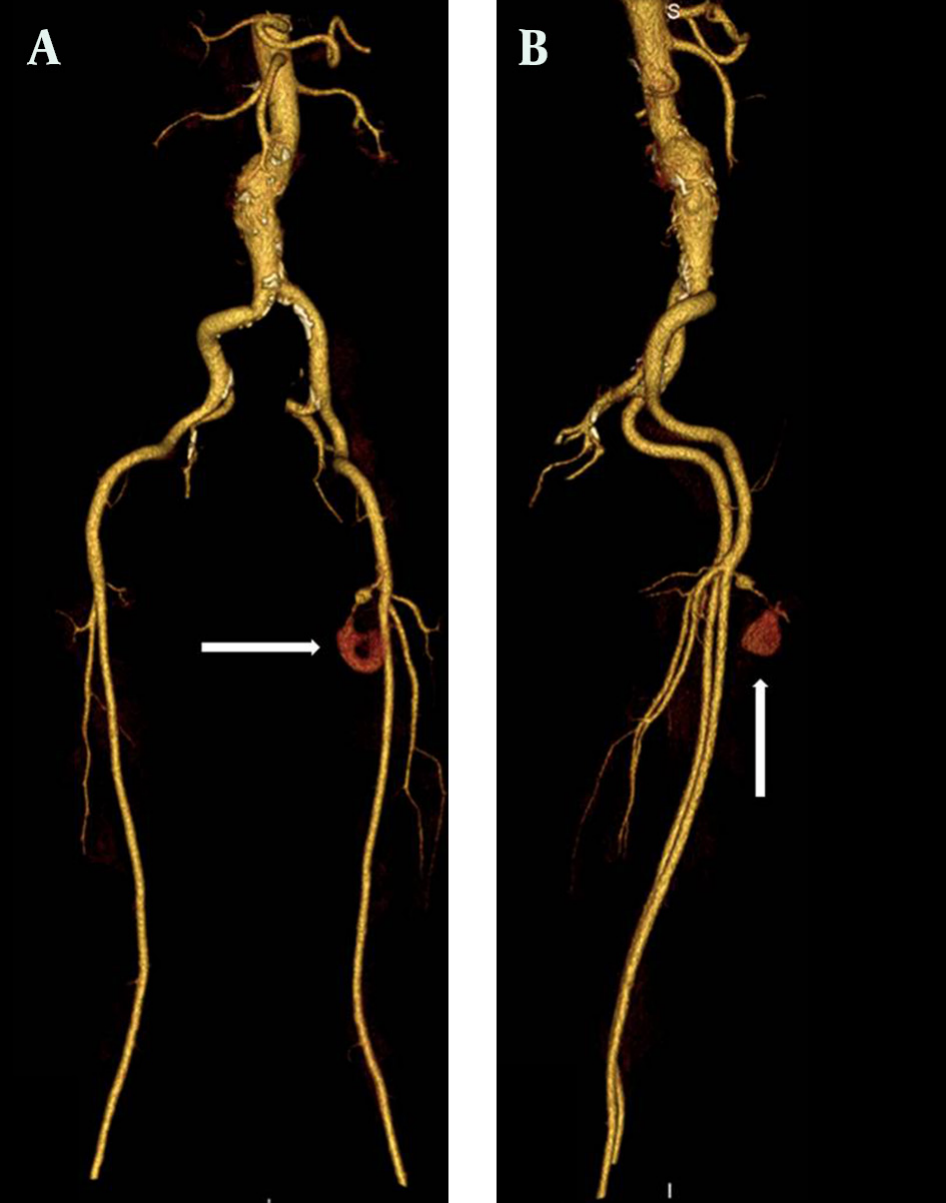
MDCTA maximum intensity projection (MIP) images. A) Anterior-posterior view B) Lateral view. Morphology of pseudoaneurysm and SEPA are clearly seen on the MDCTA MIP images (arrows).

**Figure 2.**
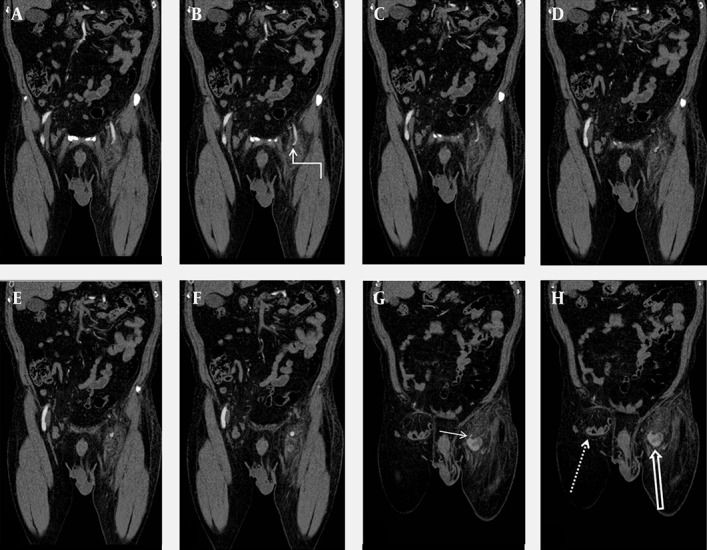
A-H) Sequential coronal MDCTA reformatted images. SEPA (curved arrow), intact femoral arteries, pseudoaneurysm (thick arrow), neck of the pseudoaneurysm (short arrow), and right inguinal hernia (dotted arrow) are clearly seen on the MDCTA images.

**Figure 3.**
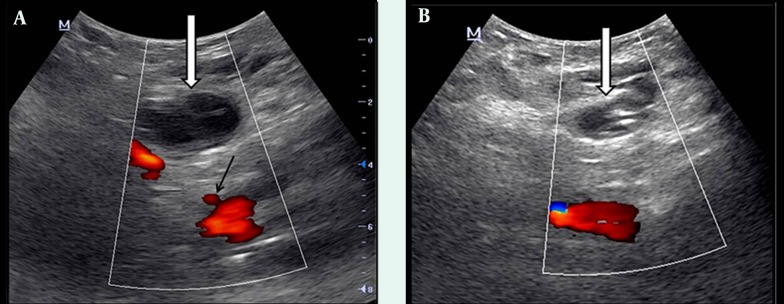
Doppler US examination images. A) Just after the second US-guided compression treatment; pseudoaneurysm is not closed (white arrow). The origin of SEPA from the common femoral artery is seen (black arrow). B) Two days after compression treatment; complete thrombosis is observed in the pseudoaneurysm (white arrow).

In 64-MDCTA (Aquilion, Toshiba Medical Systems, Tokyo, Japan) and control Doppler US examination, IPA was originated from SEPA and the common femoral artery was normal (Figure 1). In addition, an inguinal hernia containing small intestinal loops was detected in the right inguinal region (Figure 2). Iatrogenic pseudoaneurysm was 36×23 mm in size and there was a 10 mm thrombus inside it. US-guided compression treatment was applied for the second time for the patient. In US Doppler examination, IPA was completely thrombosed following 20 minutes of compression therapy. There was no recanalization observed in the control Doppler US that was performed 2 days after the first compression procedure and the patient’s complaints regressed (Figure 3). In the control Doppler US performed 3 months later, the sac of IPA was significantly regressed and the patient recovered completely without any complication.

## 3. Discussion

**Figure 4.**
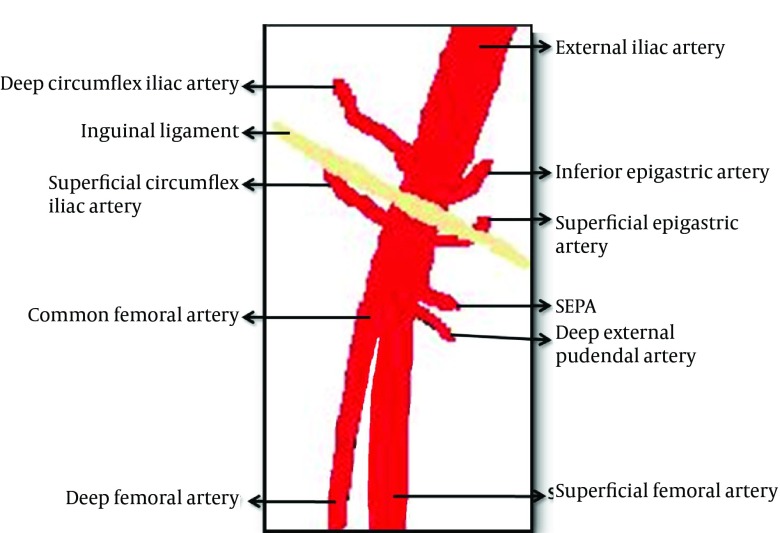
Vascular diagram of the inguinal region (7)

The incidence of IPA has been reported as between 0.05% and 7.7% according to several reports (2-6). Typical access site for diagnostic and/or therapeutic catheterization is the common femoral artery (Figure 4) (5). Therefore, IPA occurrence following catheterization is most commonly seen in the common femoral artery neighborhood. Since gray scale and Doppler US are the most important and non-invasive tools in the diagnosis of IPA, Doppler US should be the first line choice in patients suspected of IPA (4). In Doppler US examination of IPA, the following signs might be observed: hypoechoic aneurysm sac around the femoral artery and to and fro type high velocity waveform in the aneurysm sac or near the neck of the aneurysm (6).

There are different treatment modalities for the iatrogenic pseudoaeurysm of the femoral artery (3). US-guided compression treatment is an expensive, safe, and non-invasive method among these modalities (5, 6). In case this treatment fails, or when IPA exceeds a size of 4 cm, minimally invasive percutaneous treatment techniques including thrombin, saline or collagen injection, coil embolization or insertion of covered stent should be considered (4-6, 8). All of these techniques have their own disadvantages. Although minimally invasive techniques are more effective than US-guided compression; they are more expensive and could cause various complications (including thrombotic complication, bleeding, anaphylaxis and infection) (3). Moreover, surgical treatment might result in serious complications, and should be performed only when minimal invasive techniques have failed (6). For these reasons, US-guided compression was our first choice in the presented case and complete healing was achieved in two sessions.

There are various factors that have an impact on IPA development. Among these, arterial hypertension, improper puncture site, thrombolytic-anticoagulant agent, and the larger size of the introducer used are the most important ones (2). Moreover, Popovic et al. demonstrated that the access side is an important predictive factor for IPA development (2). A right-handed interventionist could have problem in directing the needle in the right position and puncturing the artery in the proper position when working on the left side of the patient, as in the presented case.

The superficial external pudendal artery is located at the level of the femoral artery bifurcation, originates from the medial side of the common femoral artery, and proceeds to the perineal region (7). In MDCTA examination, we observed that the neck of IPA originated from SEPA. Therefore, we thought that the intervention at the bifurcation level of the femoral artery leading to this situation was performed by a right-handed cardiologist from the right side with an improper angle. Detection of the puncture area of the introducer at the medial side of the neck of the femur in US examination supported our thought. In the presented case, due to inguinal hernia on the right side, the left groin had been accessed. In patients in whom therapeutic angiography will be performed from the left groin, to prevent these types of complications, arterial puncture should be done under Doppler US guidance before intervention. It was reported that this maneuver could reduce the incidence of IPA development significantly (9). Furthermore, the exact position of the needle should be determined with fluoroscopy before arterial puncture. The needle should enter the skin with a 45° angle from the conjunction of the femoral head and neck, and the femoral artery should be punctured exactly in the mid-femoral head level. In this level, the femoral artery is supported by the femoral head posteriorly; therefore, post procedure hemostasis could be easily provided with compression (5).

It has been reported that pseudoaneurysm of the pudendal artery is associated with endorectal prostate biopsy, penetrating gluteal trauma, ischial pressure wound with secondary infection, and blunt pelvic trauma (10). To the best of our knowledge, IPA of the pudendal artery due to angiographic catheterization has not been reported in the literature. In our case, the diagnosis of pudental artery IPA was made by MDCTA with 3D reconstructions. Doppler US examinations may have limitations in detecting small pseudoaneurysms of the proximal extremities and localizing the neck of the pseudoaneurysm correctly (10). MDCTA has many benefits, including being accurate, rapid, and relatively operator independent (10). Moreover, MDCTA is cheaper, less invasive with a lower complication rate when compared with conventional angiography, which is accepted as the gold standard (10). MDCTA enabled detailed diagnostic evaluation sufficient for pretreatment planning of IPA (10).

In conclusion, the incidence of iatrogenic pseudoaneurysm has increased recently due to the increased number of therapeutic catheterization and/or the improper technique. In the current patient, the pseudoaneurysm arose from a different vessel than usually seen. In patients in whom therapeutic angiography will be performed from the left groin, arterial puncture should be done under Doppler US guidance to reduce the risk. Doppler US examination can be an effective, non-invasive, and sufficient tool for IPA diagnosis and may also be part of the treatment when using the compression technique (5). In case we cannot evaluate IPA morphology, MDCTA can be used as an effective and safe technique. US-guided compression technique for IPA therapy is a noninvasive, inexpensive, easy, and well tolerable method, with low mortality and a high success rate in selected patients (5). Minimally invasive percutaneous techniques should be used when US-guided compression is unsuccessful.
